# Estrogen receptor-alpha (ER-alpha) and defects in uterine receptivity in women

**DOI:** 10.1186/1477-7827-4-S1-S9

**Published:** 2006-10-09

**Authors:** Bruce A Lessey, Wilder A Palomino, KBC Apparao, Steven L Young, Ruth A Lininger

**Affiliations:** 1 Division of Reproductive Endocrinology and Infertility, University Medical Group, Department of Obstetrics and Gynecology, Greenville Hospital System, Greenville, SC, USA; 2 Department of Obstetrics and Gynecology, University of North Carolina, Chapel Hill, NC, USA; 3 Department of Pathology and Laboratory Medicine, University of North Carolina, Chapel Hill, NC, USA

## Abstract

Endometriosis is a disorder that affects 5% of the normal population but is present in up to 40% of women with pelvic pain and/or infertility. Recent evidence suggests that the endometrium of women with endometriosis exhibits progesterone insensitivity. One endometrial protein that fluctuates in response to progesterone is the estrogen receptor-alpha (ER alpha), being down-regulated at the time of peak progesterone secretion during the window of implantation. Here we demonstrate that the biomarker of uterine receptivity, beta 3 integrin subunit, is reduced or absent in some women with endometriosis and that such defects are accompanied by inappropriate over-expression of ER alpha during the mid-secretory phase. Using a well-differentiated endometrial cell line we showed that the beta 3 integrin protein is negatively regulated by estrogen and positively regulated by epidermal growth factor (EGF). By competing against estrogen with various selective estrogen receptor modulators (SERMs) and estrogen receptor agonists and antagonists, inhibition of expression of the beta 3 integrin by estrogen can be mitigated. In conclusion, we hypothesize that certain types of uterine receptivity defects may be caused by the loss of appropriate ER alpha down-regulation in the mid-secretory phase, leading to defects in uterine receptivity. Such changes might be effectively treated by timely administration of the appropriate anti-estrogens to artificially block ER alpha and restore normal patterns of gene expression. Such treatments will require further clinical studies.

## Introduction

Uterine receptivity is regulated by the cyclic secretion of ovarian steroids as a consequence of follicular development and subsequent ovulation. As first described in the uterus by its ability to bind estrogen [1], the classic estrogen receptor (ER-alpha), increases in response to estrogen during the proliferative phase, and is diminished in response to progesterone during the secretory phase [2]. Progesterone receptors (PR) undergo similar changes; we and others previously reported that expression of both ER-alpha and PR are down-regulated at the time of implantation, particularly in the epithelial compartment [3-5]. While progesterone is an effective anti-estrogen, its role is to reduce ER-alpha concentration rather than to act as a competetive inhibitor of E2 binding, thereby rendering the endometrium resistant to estrogen during the window of implantation. This phenomenon has now been observed in several mammalian species [6-8].

While steroid hormones were classically described as stimulating gene expression, we now recognize from genomic studies that steroid hormones can also inhibit specific genes [9,10]. In endometrium, the decline in ER-alpha may be a critical event, releasing an inhibitory influence on specific genes and providing a signal for endometrial receptivity to commence. The highly specialized and specific mid-secretory repertoire of endometrial gene expression coincides with a reduction in the expression of ER-alpha [10]. Based on such studies, we now suggest that the combination of progesterone action and coincident estrogen withdrawal is required to stimulate key implantation-specific events in the mid-secretory phase of the menstrual cycle.

Early implantation is a complex process involving synchronous and complementary events on both maternal and embryonic surfaces. It is now well-established that many proteins are expressed specifically at the time when embryos implant [11,12]. The timing of implantation has now been firmly established, occurring around 7 to 10 days after ovulation, corresponding to days 21 to 24 of an idealized 28 day menstrual cycle [13]. Integrins are one of many endometrial proteins that turn on or off around this time in a woman's cycle [14,15], and the study of their patterns of expression has yielded substantial information about the factors that regulate endometrial receptivity in the human [16,17]. One of these integrins is the alpha v/beta 3 integrin that is expressed on the luminal and glandular epithelium of the endometrium. Its appearance at the time of implantation and its aberrant expression in a variety of settings including luteal phase defect, endometriosis, polycystic ovarian syndrome (PCOS) and tubal disease suggests a critical role for this protein [18-21]. We recently demonstrated that the reduction in endometrial beta 3 integrin expression in PCOS was associated with elevated expression of ER-alpha and an over-expression of steroid receptor coactivators [20]. In the present report we examine the basis for this effect of estrogen on this particular marker of uterine receptivity and study women with endometriosis as another group who appear to inappropriately express high levels of ER-alpha during the window of implantation.

## Materials and methods

### Patient samples

For these studies we used endometrial samples obtained from 38 volunteers who had normal menstrual cycles and proven fertility at various times in the menstrual cycle (proliferative, n = 7; early secretory = 22; mid-secretory, n = 13; late secretory = 6). Some of these samples were obtained in women undergoing bilateral tubal ligation, each found to be free of endometriosis at laparoscopy. The 13 mid-secretory samples were obtained in fertile controls that were recruited and paid for their participation. In addition, 19 mid-secretory endometrial biopsies were obtained from women with infertility that subsequently had endometriosis documented by laparoscopy. Each of these endometrial samples were selected because they lacked epithelial expression of the alpha v/beta 3 integrin, despite having normal, "in phase" histologic changes consistent with day 21 to 24 of the menstrual cycle, according to the dating criteria of Noyes et al [22]. All mid-secretory samples were obtained and timed to the urinary LH surge (Ovuiquick, Quidel, San Diego, CA). All samples were formalin-fixed and paraffin embedded. Histologic stage (endometrial dating) was assigned by two of the investigators (RAL and BAL). Samples were eliminated from further consideration if there was evidence of histologic delay that exceeded 3 days compared to the expected endometrial dating based on urinary LH testing. The acquisition and use of human tissue for this study was approved by the Institutional Review Board at the University of North Carolina at Chapel Hill and Greenville Hospital System, Greenville, SC.

### Immunohistochemistry

Immunostaining for ER-alpha was performed on 5-to 6-μ sections of formalin fixed, paraffin-embedded endometrial biopsies using the Vectastain Elite ABC kits (Vector Laboratories, Inc.,Burlingame, CA). Diaminobenzidine (Sigma, St. Louis, MO) was used as the chromagen. Tissue sections were deparaffinized in xylene and hydrated in a series of graded ethanols. Following a rinse in PBS, pH 7.4, endogenous peroxidase activity was quenched upon incubation for 30 min with 0.3% H2O2 in absolute ethanol, followed by a 10-min rehydration in PBS. Tissue sections were heated in a microwave for 12 min in antigen retrieval solution (10 mm citrate buffer) before incubation with primary antibody. After initial incubation with blocking serum for 30 min at room temperature (4% normal goat serum), sections were incubated with mouse monoclonal antibodies against human ER-alpha (Novocastra, New Castle upon Tyne, UK) overnight at 4 C. Negative controls were analyzed on adjacent sections incubated without primary antibody. Each primary antibody was serially diluted to achieve maximum sensitivity and specificity. A PBS rinse was followed by treatment of secondary antibody consisting of biotinylated goat antimouse antibody for 30 min. After this incubation, sections were washed and incubated with avidin:biotinylated horseradish peroxidase macromolecular complex for 60 min. Visualization of peroxidase was carried out by adding diaminobenzidine and incubated for 10 min to complete the reaction. As a final step, sections were counterstained with hematoxylin, dehydrated in a graded series of ethanols, cleared with xylene, and a coverslip placed over Permount for evaluation by light microscopy. The resulting staining was evaluated using a Nikon microscope (Tokyo, Japan), by a single observer (B.A.L.) without knowledge of the specimen's identity. Assessment of staining intensity and distribution was made using the semi-quantitative histologic score (HSCORE) system in the glandular and luminal epithelium and in the stromal compartment. HSCORE was calculated using the following equation: HSCORE: ∑ Pi (i + 1), where i represents the intensity of staining ranging from 1, 2, or 3, (representing weak, moderate or strong, respectively) and Pi representing the percentage of stained endometrial stromal and epithelial cells for each intensity, varying from 0–100%.

### Cell Culture and Western Blot Analysis

Ishikawa cells, the well differentiated endometrial carcinoma cell line, were obtained from Dr. M. Nishida (Kasumigaura National Hospital, Tsuchiura-shi, Ibaraki-ken, Japan) and maintained in MEM (Life Technologies, INC., Grand Island, NY) containing 5% charcoal stripped fetal bovine serum (FBS), 1% pencillin-streptomycin (Life Technologies, INC., Grand Island, NY) and 2 mM L-glutamine (Life Technologies, INC., Grand Island, NY) at 37°C in the presence of 5% CO2. The culture medium was changed every 2–3 days. This cell line contains a functional estrogen receptor and as evident by estrogen-induced alkaline phosphatase enzyme activity bioassay, has been successfully used as a measure of estrogenic activity in these cells [23,24]. Cells were treated in phenol red-free DMEM/F12 media with 0.5% heat-inactivated charcoal-stripped FBS, 1% pencillin-streptomycin and 2 mM L-glutamine. Cells plated onto 100 mm dishes were exposed to medium alone (control) or medium containing 17β-estradiol (E_2_; 10^-8 ^mol/L; Sigma) and/or epidermal growth factor (EGF; 10 ng/ml) individually or in combination with the estrogen antagonist, ICI 182780 (10^-6 ^mol/L; Zeneca Pharmaceuticals, Wilmington, DE) or C225 antibody (30 nM/ml; monoclonal antibody that neutralizes the EGF receptor, provided by Imclone, Inc. (New York, NY). In other experiments cells were treated with and with E2 (10^-8 ^mol/L; Sigma), estriol (E3; 10^-8 ^mol/L; Sigma) or equimolar concentrations (10^-8^M) of selective estrogen receptor modulators (SERMs: Clomiphene Citrate, Tamoxifen, Raloxifene) The time of exposure in each experiment was 72 h.

For immunoblotting of Ishikawa cell extracts, cells treated as described above were harvested individually and cell pellets obtained from the respective treatments were resuspended in RIPA extraction buffer (10 mM Tris-HCl [pH 8.0], 10 mM EDTA [pH 8.0], 150 mM NaCl, 1% NP-40, and 0.5% SDS) containing protease-inhibitor cocktail (Boehringer Mannheim, Indianapolis, IN). After centrifugation at 10 000 × g for 10 min, protein concentrations were determined using the Bio-Rad protein assay kit (Bio-Rad Laboratories, Hercules, CA). Total proteins (100 μg from each treatment) were denatured in Laemmli buffer, electrophoresed on 10% SDS-containing polyacrylamide gel and transferred to nitrocellulose membrane. Blots were blocked for 1 h in TBST (20 mM Tris [pH 7.5], 137 mM NaCl, and 0.4% Tween-20) containing 10% nonfat dry milk. Blots were washed twice for 5 min each time in TBST, then incubated overnight with a 1:1000 dilution of monoclonal antibody against alpha v/beta 3 integrin (SSA6; a generous gift from James Hoxie, University of Pennsylvania, Philadelphia, PA) while rocking at 4°C. The blots were then washed twice for 30 min each time with TBST, followed by incubation for 1 h at room temperature with peroxidase-conjugated anti-mouse IgG secondary antibody while rocking. After washing twice for 30 min each time with TBST, the immunoreactive protein complexes were detected by enhanced chemiluminescence (Amersham Pharmacia Biotech Inc., Piscataway, NJ). The results depicted are representative of 2 independent experiments.

### Statistical Analysis

Comparisons between treatment groups were analyzed using ANOVA with Scheffe's correction for multiple comparisons using 95% confidence interval (p < 0.05). Analysis was performed on a Dell PC with GraphPad Prism 4 software.

## Results

**Figure 1:**
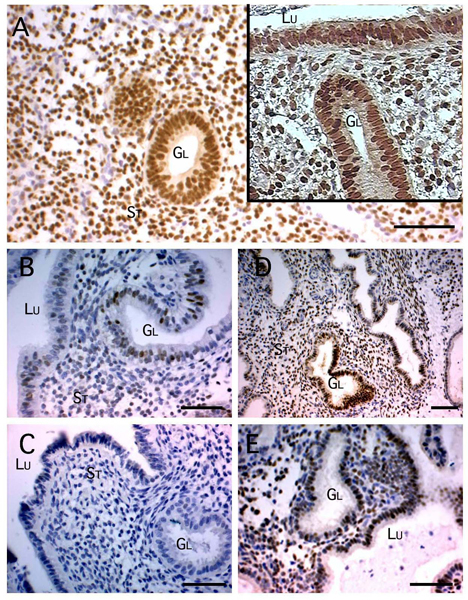
Immunohistochemistry of ER-alpha in normal cycling women from the proliferative with inset showing luminal epithelium (A), early secretory (B) and mid-secretory (C) endometrium. Other samples from the mid-secretory endometrium from women with endometriosis shows increased and inappropriate expression of the ER-alpha protein in glandular and stromal compartments (D and E) compared to normal mid-secretory endometrium (C). Bar = 50 μm.

**Figure 2:**
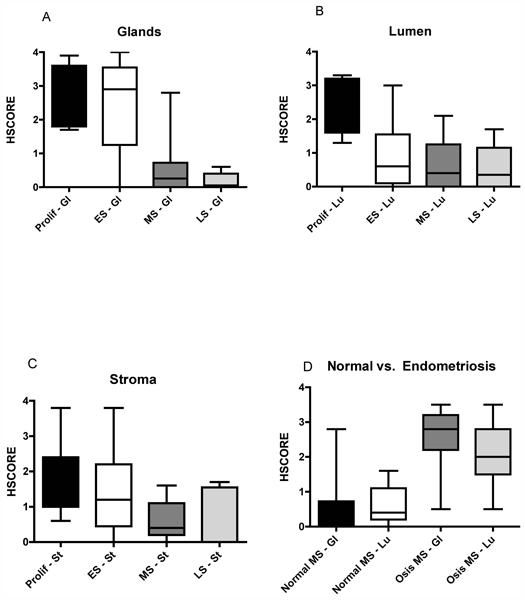
Box and whisker plots are shown depicting the semi-quantitative immunohistochemical staining (HSCORE ± SD) for ER-alpha in normal women throughout the menstrual cycle in proliferative, early, mid and late secretory glandular (A) and luminal (B) epithelium and in stroma (C). In a separate analysis, glandular and luminal ER-alpha HSCOREs were compared between normal fertile mid-secretory endometrium and similar samples in women with endometriosis and suspected defects in uterine receptivity (D). Significance was based on ANOVA with Bonferroni correction; * = p < 0.01, ** = p < 0.001. Abbreviations in the figure: Prolif = proliferative phase, ES = early secretory, MS = mid-secretory, LS = last secretory, gl = glandular epithelium, St = stroma, LU = luminal epithelium, Osis = endometrium from women with endometriosis. Note that the horizontal bars in the box and whisker plots designate the 10^th^, 25^th^, 50^th^, 75^th^, and 90^th ^percentile distribution of the data and standard error bars are not shown in this figure.

The pattern of expression for ER-alpha changed throughout the menstrual cycle, as judged by immunohistochemistry. As shown in Figures 1A–C and 2A–C, the temporal and spatial pattern of ER-alpha was dynamic, with increased expression in glandular, luminal, and stromal compartments in the proliferative and early secretory phases, and a subsequent decline in the mid to late-secretory phases. Based on HSCORE determinations, the expression of ER-alpha in all three cell types was significantly lower in the secretory than the proliferative phase. Luminal epithelium exhibited reduced ER-alpha immunostaining relative to glandular epithelium. Stromal cells also were devoid of ER-alpha immunostaining during the time of maximal uterine receptivity in normal fertile samples. Later in the secretory phase, ER-alpha immunostaining returned to the stromal compartment but little if any immunostaining was observed in the epithelium in normal fertile controls.

Women with endometriosis who exhibited an aberrant lack of the alpha v/beta 3 integrin, as a group exhibited a significantly higher level of ER-alpha expression in epithelial cells compared to normal fertile controls. Examples of this are shown in Figure 1D and 1E. A comparison of ER-alpha staining intensity and distribution (HSCORE) is also shown in Figure 2D comparing glandular and luminal ER-alpha distribution only. Note the higher levels of ER-alpha immunostaining in both cell types in women with endometriosis and suspected defects in uterine receptivity compared to normal women during the mid-secretory phase.

**Figure 3:**
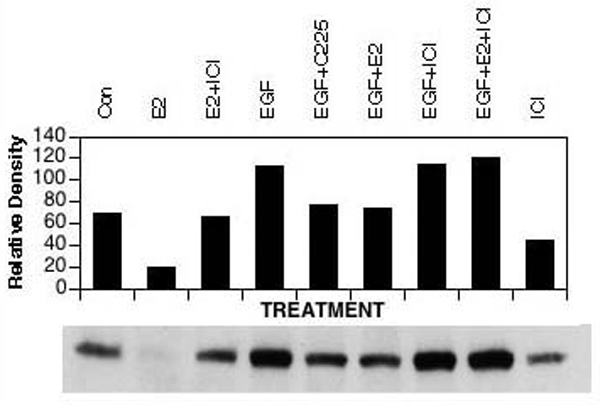
Western blot for beta 3 integrin expression in Ishikawa cells treated with no treatment (con), or three days of estradiol (E2; 10^-8^M), estradiol plus the 10^-6 ^M pure antagonist ICI 182,780 (E2 + ICI), epidermal growth factor (EGF; 10 ng/ml), EGF plus the EGF receptor antagonist antibody C225 (EGF + C225), EGF + ICI, EGF + E2 + ICI and ICI alone.

To understand the mechanism of the action of estrogen on beta 3 integrin expression, we used the hormone responsive, well-differentiated, Ishikawa cell line. These cells mimic the pattern of gene expression for endometrial epithelium and provide a model to study factors that regulate specific genes in human endometrium [23,25-29]. As shown in Figure 3, using western blotting analysis, we show that estrogen strongly inhibits the expression of the beta 3 integrin subunit in Ishikawa cells, while EGF significantly increases its expression. Blockade of the EGF receptor with a neutralizing antibody (C225) mitigates this effect by EGF. Likewise, E_2 _counteracts the stimulatory effect of EGF on beta 3 expression. The absolute estrogen antagonist, (ICI 182780) released the inhibitor effect of estradiol on beta 3 integrin expression, as well. This experiment suggests that the beta 3 gene product is regulated in endometrial epithelium by dual mechanisms that includes both stimulatory and inhibitory components and that estrogen inhibition appears to be dominant over EGF stimulation.

**Figure 4:**
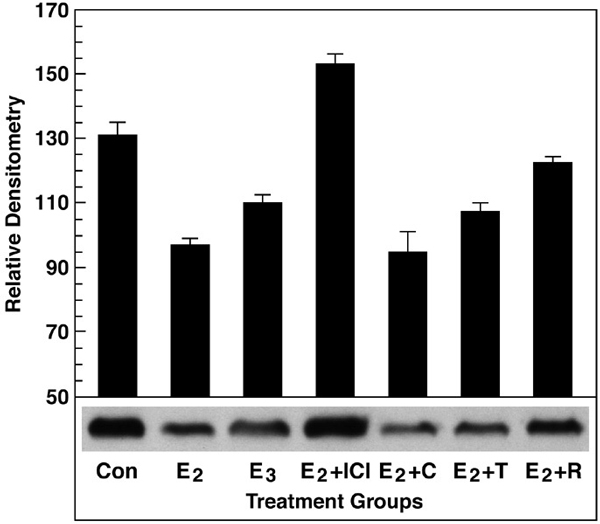
Western blot showing the effect of estrogen and anti-estrogens on the inhibition of the beta 3 integrin subunit in Ishikawa cells. In control cells, the beta 3 integrin was expressed while 10 ^-8 ^M estradiol reduced that expression significantly. E3 (estriol) was less potent than estradiol (E2) at inhibiting beta 3. Of the SERMS, in cells treated with E2 plus equimolar concentrations of other anti-estrogens and SERMs, a differential competitive antagonism occurred with the most potent antagonism by ICI 182,780. Raloxifene had an anti-estrogen effect on this well-differentiated endometrial cell line as well. Con = Control; E2 = estradiol; E3 + estriol; ICI = ICI 182,780, C = clomiphene citrate; T = tamoxifen; R = raloxifene.

If inhibitory factors block the expression of beta 3 integrin, we next asked how different estrogens or anti-estrogens might compare as potential treatments for loss of the beta 3 integrin. To examine the effect of various selective estrogen receptor modulators to reverse this inhibitor effect of estrogen, we pretreated Ishikawa cells with estradiol and then added equimolar quantities of either the weak estrogen (estriol; E3), the SERMs clomiphene citrate, tamoxifen, raloxifene and the anti-esrogen ICI 182780. As noted in Figure 4, tamoxifen and clomiphene citrate did little to restore normal beta 3 integrin expression. ICI 182780 was the most potent anti-estrogen in terms of reversing inhibition of beta 3 protein. Surprisingly, raloxifene was a fairly potent anti-estrogen in the endometrial cells, as it induced reasonable inhibition of the negative effect of estradiol on beta 3 integrin expression in Ishikawa cells.

## Discussion

Estrogen action has traditionally been considered mitogenic and stimulatory in most target cells. During the proliferative phase, estrogen stimulates a large number of genes including c-fos and c-jun, cell cycle proteins and the estrogen receptor itself [30,31]. Exposed to unopposed estrogen, the endometrium undergoes hypertrophy and hyperplasia and eventually risks becoming neoplastic in the absence of cyclic progesterone exposure. As shown in the present study, estrogen appears to inhibit expression of specific proteins, a role we would argue is confined to select genes that are expressed during the window of implantation. While estrogen is a major hormone in the proliferative phase, it was previously argued that estrogen is unnecessary for the normal development of the secretory phase endometrium [32]. In our view, the disappearance of ER-alpha at the time of implantation as seen in most mammalian species, argues that continued ER-alpha expression may be detrimental to the development of uterine receptivity, or at least a sign of other dysfunction in the endometrial cycle.

Based on expected steroid receptor changes, the loss of ER during the mid-secretory phase provides a time in the cycle when mitoses cease and implantation occurs. In this study we documented, again, this decline in ER-alpha that has previously been described [3,4]. Such changes in steroid receptors may account for the four stages of the endometrial cycle as defined by recent DNA microarray studies [10]. In the first, estrogen works alone to stimulate ER-alpha and PR isoforms and to increase expression of a host of genes necessary for cell division and tissue growth. With ovulation, in the early secretory phase, progesterone acts to halt cell division and to induce secretory changes. In the third (mid-secretory) phase, ER-alpha largely disappears, providing for the first time, the opportunity for progesterone to act alone, specifically targeting the stroma [3]. The stroma contributes paracrine activity in response to progesterone, stimulating epithelial gene expression indirectly [16]. During the late luteal phase, ER-alpha appears to return to the stromal compartment, reflecting the time of sloughing and regeneration that will soon follow if pregnancy does not occur.

The elevation in ER-alpha that we describe in this study, corresponds to the lack of beta 3 integrin in selected patients with endometriosis. We described similar findings in women with polycystic ovarian syndrome (PCOS). This is another condition associated with infertility and pregnancy wastage. While ER-alpha is elevated in PCOS and beta 3 levels are low, the pathophysiology may be quite different between the two diagnoses. In the former, chronic estrogen may alter the complement of steroid receptor coactivators that are present; in endometriosis, elevations in aromatase have been described, possibly raising the local production of estrogen. Alternatively, faulty progesterone receptors have been reported in this disorder. In this study, we document that ER-alpha is increased in women with suspected defects in uterine receptivity lacking the EGF stimulated and estrogen inhibited gene, beta 3.

We suspect that there will be many genes other than beta 3 integrin subunit that are regulated in concert during implantation in response to EGF and the loss of ER-alpha. There are likely to be many other endometrial genes that are inhibited by E2 and, like the beta 3 integrin protein, are only expressed when ER-alpha is down-regulated around the mid-secretory phase. Conversely, any failure to down-regulate ER alpha in infertile women may predispose to altered gene expression, including failure to express essential proteins associated with uterine receptivity [19]. Such defects have been described in the endometrium of women with endometriosis [33,34], luteal phase defect (*LPD*), and polycystic ovarian syndrom (PCOS) [19,20,35], each of which is associated with either infertility or pregnancy loss.

What might account for the aberrant down-regulation of ER-alpha by the time of implantation in all of these different disorders? In LPD, the timing of ER-alpha down-regulation is delayed, perhaps due to inadequate progesterone levels in the serum [36]. For endometriosis patients, it is clear that the endometrium of women with endometriosis is different from normal endometrium [33,34,37]. An important difference between eutopic endometrium from women with and without endometriosis is also inappropriate expression of aromatase [38,39]. If aromatase increases local estrogen production within the endometrium, this might alter the balance between estrogen and progesterone and could therefore account for the elevation in ER-alpha observed in this study. Alternatively, increased IL-6 has been demonstrated in the endometrium of women with endometriosis. IL-6 was recently shown in breast cancer cells to activate ER and this could lead to increased ERα expression [40,41]. Whether this effect is mediated through modulation of aromatase is not clear. In endometriosis patients, defects in the progesterone receptor have been postulated [37]. Loss of progesterone activity may result in the persistence of ER-alpha, since down-regulation of ER is one of the primary actions of progesterone. Finally, in PCOS, we suspect that elevated ER-alpha levels are also associated with a decrease in uterine receptivity. In this case, however, the failure to down-regulate ER-alpha may be due to a hypersensitivity to estrogen, as evidenced by the over-expression of steroid receptor coactivators and ER-alpha itself [20].

Thus, uterine receptivity defects may represent a common pathway for many women with infertility. The presence of ER-alpha in mid-luteal endometrium may be the best biomarker of a dysfunctional endometrium. Treatment of such defects might be designed to block the estrogen receptor or to correct the underlying cause of its over-expression. In women with elevated ER-alpha during the window of implantation, short-term treatment with anti-estrogens may prove useful. If Raloxifene were used, for example, its anti-estrogenic blockage of ER-alpha activity may result in a normalization of biomarkers of uterine receptivity. To our knowledge, no one has suggested such a novel use of anti-estrogens, to stimulate uterine receptivity. Alternatives to anti-estrogens might also include the use of stronger progestins or the use of aromatase inhibitors. Only appropriately designed clinical trials will we be able to address these possibilities. Given the large number of women that might be affected, (e.g. PCOS, endometriosis or LPD) the potential benefits are substantial. A simple and direct treatment to restore normal uterine receptivity in the cycle of conception would truly be an enormous advance in our approach to women with implantation failure.
